# Case Report of Undifferentiated Endometrial Sarcoma in Association with Osteoclast-Like Giant Cells

**DOI:** 10.4061/2011/629840

**Published:** 2011-03-30

**Authors:** Svetoslav Bardarov, Vadim Khachaturov, Patel Kirtesh, Elpidio Jimenez

**Affiliations:** Department of Pathology, 97 Amity Street, Brooklyn, NY 11201, USA

## Abstract

We describe the clinical, gross and microscopic features of undifferentiated uterine stromal sarcoma associated with osteoclast-like giant cells. A case of low-grade endometrial stromal sarcoma is already described in association with osteoclast-like giant cells; however, the current case differs in that the tumor was a high grade and did not show any evidence of smooth muscle or epithelioid differentiation and was shown to be strongly positive for CD10 and focally for WT-1 and Inhibin supporting an endometrial stromal origin. The associated osteoclast-like giant cells were abundant, evenly distributed within the tumor and showed strong positivity for CD68. Interestingly, rare (less than 2%) giant cells also showed weak cytoplasmic positivity for b-hCG. The tumor infiltrated deep into the myometrium and had marked lymphovascular invasion. Although the regional lymph nodes and peritoneal washings were negative, the lesion showed a highly aggressive clinical course. Despite treatment, the tumor disseminated within the abdominal cavity and lungs and ultimately led to the patient's demise within 9 months of the diagnosis.

## 1. Introduction

Endometrial stromal sarcomas are rare tumors comprising approximately 1-2% of all tumors of the uterine corpus. Larson et al. [1] showed that the mean age of presentation for low-grade endometrial stromal sarcoma was approximately 47.2 years and for high-grade endometrial sarcoma about 50 years with a most common presenting symptom of menorrhagia. Classically, the distinction between low-grade and high-grade endometrial stromal sarcoma was based on mitotic count; however, since high-grade endometrial stromal sarcoma bears no histologic resemblance to low-grade endometrial stromal sarcoma, it has been proposed that high-grade tumors be renamed to undifferentiated endometrial sarcoma. WHO defines this sarcoma as: “High-grade endometrial stromal sarcoma that lacks specific differentiation and bears no histologic resemblance to endometrial stroma” [2]. This undifferentiated tumor presents with increased mitotic count, more than 10 mitoses/10 HPF and show an aggressive clinical course. Death occurs from tumor dissemination within 3 years after the diagnosis. Undifferentiated endometrial sarcomas display morphologic diversity which may be the source of diagnostic difficulties. Probably the very first case of poorly differentiated endometrial stromal sarcoma with osteochondromatous differentiation and benign appearing giant cells is documented approximately 30 years ago by Evans in 1982 [3] however this neoplasm was not described in detail. In 2005 Fadare et al. [4] described a unique case of a low-grade endometrial stromal sarcoma with smooth muscle differentiation which was associated with the presence of osteoclast-like giant cells; however the first case of true giant cell “nonleiomyomatous” tumor of the uterus was described in 2007 by Skubitz and Carlos Manivel [5]. The authors describe a case of a 55-year-old female who was found to have a uterine neoplasm with multiple lung nodules. The neoplasm was composed of atypical plum mononuclear cell and numerous osteoclast-type giant cells. The neoplastic mononuclear cells demonstrated positive Vimentin, focal CD10 stains as well as week actin stains; however, they tested negative for Desmin and Smooth muscle myosin arguing against the smooth muscle origin of this tumor. The authors classified this tumor as a giant cell tumor of the uterus similar to the giant cell tumor of bone. Although osteoclast-like giant cells have been described in a variety of other tumors including leiomyosarcoma of the uterine corpus and giant cell tumor of tendon sheath, this was the first reported case describing osteoclast-like giant cells in association with high-grade endometrial stromal sarcoma. 

In this paper we report and characterize a case of undifferentiated endometrial sarcoma with osteoclast giant cells. We also elaborate on the cytological, gross and microscopic pathological findings, including the immunohistochemical profile of this unique entity.

## 2. Materials and Methods

The specimen was fixed in 10% formalin and processed for histologic examination using conventional methods. 

Immunohistochemical analysis was performed using formalin-fixed, paraffin-embedded sections. The avidin-biotin peroxidase complex technique and the peroxidase-antiperoxidase techniques were employed. Commercially available antibodies used in this study are summarized in Table 1. Appropriate positive and negative control experiments were also performed.

## 3. Results

### 3.1. Report of Case

The patient is a 46-year-old female who presented in Feb 2009 to the Long Island College Hospital with menorrhagia, lower abdominal cramping, anemia and vaginal bleeding. Sonogram was performed and demonstrated a 13.5 × 10.1 cm uterus with multiple fibroids. The patient underwent an endometrial biopsy which was nondiagnostic and showed mostly blood and fragments of unremarkable endocervical and squamous epithelium. The recurrent vaginal bleeding prompted a private gynecologic office visit for uterine artery embolization. Since the procedure failed to stop the bleeding, she was readmitted to the hospital in May 2009. A ThinPrep Pap smear performed during this admission showed evidence of a high-grade sarcoma. A subsequent endometrial biopsy confirmed the cytological diagnosis of high-grade sarcoma and also demonstrated a presence of numerous osteoclast-like giant cells.

At the end of May 2009, the patient underwent a total hysterectomy with bilateral salpingo-oophorectomy with lymph node dissection. The surgery was uneventful.

### 3.2. Radiologic Findings

CT of the abdomen and pelvis was performed from the dome of the diaphragm to the pubic symphysis after administration of oral and IV contrast via 5 mm reconstruction.

**Figure 1:**
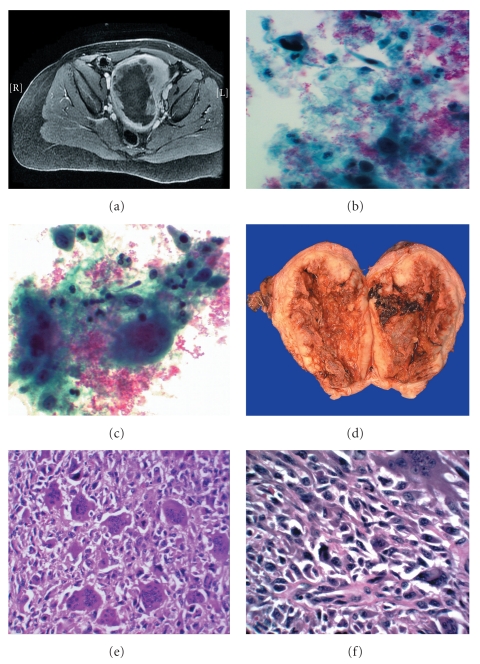
(a) MRI (T1 weighted, after Gadolinium injection): Enlarged uterus with central hypodense area. (b) ThinPrep Pap smear showing numerous spindle cells with marked anysocytosis, anisonucleosis and chromatin condensation. The background shows granular tumor diathesis and blood (Pap, 400x). (c) ThinPrep Pap smear: Multinucleated giant cells in association with single spindle and more epithelioid appearing malignant cells (PAP, 400x). (d) Hysterectomy gross specimen showing anterior and posterior endometrial cavities occupied with tumor with central hemorrhagic area. (e) Histologic sections showing high-grade sarcoma with osteoclast-like giant cells, numerous mitotic figures and individual cell apoptosis (H&E, 200x). (f) High power view of histological sections showing undifferentiated endometrial sarcoma with marked cellular pleomorphism and atypical mitoses (H&E, 400x).

The gynecological structures demonstrated an enlarged myomatous uterus with areas of low attenuation which were interpreted as representing areas of necrosis. The estimated anterior-posterior diameter dimension of this large hypodensity was 10.8 cm (Figure 1(a)). Subsequent MRI revealed an intramural lesion with a large submucosal component which distorted the underlying endometrium.

### 3.3. Cytology Findings

The ThinPrep Pap smear, obtained prior to the endometrial biopsy, was paucicellular showing predominantly blood and granular fibrinous background. The majority of the malignant cells were clustered at the periphery of the slide leaving an empty central area. The tumor cells, round to spindle, were intermediate in size, with nuclei measuring approximately 15–25 microns. The cytoplasm was abundant and finely granular. The nuclei were positioned eccentrically with coarse to “pitch dark” chromatin and inconspicuous nucleoli. There was marked nuclear atypia and variable anisocytosis with large, hyperchromatic nuclei and marked nuclear membrane irregularities. Numerous abnormal mitotic figures were also found. Individual cell apoptosis and cellular mummification was readily apparent and there was abundant finely granular tumor diathesis (Figure 1(b)). Nuclear palisading, rosetting or myxoid changes were not observed. Numerous osteoclast-like giant cells were identified intimately mixed with the tumor cells. These giant cells were found to have and abundant cytoplasm with well-defined cytoplasmic borders and approximately 10–20 nuclei some of which show prominent nucleoli (Figure 1(c)).

### 3.4. Gross Anatomic Findings

The specimen received for pathologic evaluation consisted of an intact uterus and weighing 530 g with attached bilateral adnexa. The uterine serosal surface was smooth with no obvious tumor implants or perforation. Opening the endometrial cavity revealed a 12.5 × 6.0 × 3.5 cm necrotic polypoid tumor, occupying the anterior and posterior endometrium. The lesion was limited to the corpus and did not extend to the endocervix. It grossly invaded more than one half of the myometrium approaching the serosal surface (Figure 1(d)). The cut surface of the tumor was yellow, fleshy, centrally hemorrhagic and necrotic with multiple fibrotic/desmoplastic areas. The attached fallopian tubes and ovaries did not show any evidence of tumor involvement.

### 3.5. Light Microscopic Findings

The histologic sections revealed a highly pleomorphic spindle cell neoplasm, infiltrating deep into the myometrium. A second population of round and polygonal cells was also observed with high mitotic index (50–100/10 HPF), individual cell apoptosis and necrosis (Figure 1(e)). This high-grade sarcoma also showed rare extremely pleomorphic cells with abundant deeply eosinophilic cytoplasm and hyperchromatic nuclei with nuclear sizes exceeding 50 microns (Figure 1(f)). A lymphovascular invasion was also identified.

Additionally, numerous osteoclast-like giant cells were observed, evenly distributed within the tumor. Similar to the cytological finding on ThinPrep Pap smear they contained numerous (10–20) bland nuclei, some of which showed prominent nucleoli. These nuclei were partially overlapping and clustered at the center of the cytoplasm.

The tumor was not found to extend into the endocervical stroma or glands and the regional lymph nodes did not show evidence of metastatic disease. The peritoneal washings were also negative.

Immunohistochemical analysis of the tumor revealed strong positivity for CD10 and focal positivity for Inhibin and WT-1. The tumor cells tested negative for Myoglobin, Caldesmon, Desmin, Myogenin, AE1/3, S-100, Calretinin and CD56. The multinucleated osteoclast-like giant cells were found to be strongly positive for CD68 (Table 1). Some of these cells (less than 2% of the total osteoclast-like giant cell population) also exhibited a weak 2+ cytoplasmic stain for b-HCG; however typical trophoblastic cells were not identified. While this observation is of little clinical significance it is important not to misdiagnose this entity as an epithelial tumor with trophoblastic giant cells especially when dealing with limited material. The Estrogen and Progesterone receptors as well as CD117 (c-kit) were negative.

## 4. Discussion

Endometrial stromal tumors are rare mesenchymal neoplasms which occur in females between ages of 40 and 60 years often presenting with menometrorrhagia as most common symptom [1, 6]. These rare neoplasms were described and characterized in 1966 by Norris and Taylor [7] who classified them as benign stromal nodules or malignant sarcomas, depending on the degree of the mitotic activity. Currently, malignant stromal neoplasms are defined by the WHO as high-grade endometrial sarcomas that lacks specific differentiation and bears no histologic resemblance to the endometrial stroma [2]. 

Evans was the first one to document the osteochondromatous differentiation in endometrial stromal sarcoma [3], and additionally, in 2005, Fadare et al. [4] described a case of a 70-year-old woman who underwent total abdominal hysterectomy for recurrent polyps and an enlarging uterine mass. The light microscopic evaluation showed that the tumor was composed of short fusiform cells with minimal cytoplasm and a rich vascular network composed of small capillaries. The tumor was also shown to have focal smooth muscle differentiation and mitotic activity of 3–5/10 HPF. This low-grade endometrial stromal sarcoma was unique because it was the first endometrial stromal tumor that was associated with the presence of osteoclast-like giant cells.

The osteoclast-like giant cells share a morphologic and immunohistochemical similarities to regular osteoclast cells [8] and have been described in association with a variety of other tumors including poorly differentiated liver and pancreatic carcinoma [8–11]. They are believed to be of histiocytic origin, as proven by their immunophenotype and ultrastructure [4, 8, 9]. The exact relationship between these cells and the high-grade tumors is currently unknown. One theory suggests that these cells could represent transformed tumor cells. This theory was supported by molecular studies of giant cell tumors of pancreas and liver showing the same K-*ras* mutations in the tumor cells and their precursor lesions [10]. Alternative theory which gained more support is that these osteoclast-like giant cells are stromal in origin and represent a reactive host response. The fact that these cells were found to be diploid while the adjacent tumor cells were found to be aneuploid or hyperploid [11] further supports this theory.

In contrast to the low-grade endometrial sarcoma described by Fadare et al., the case presented in this paper is an aggressive sarcoma. Necrosis was readily appreciated, the mitotic counts were high, 50–100/10 HPF, and the tumor infiltrated diffusely into the myometrium approaching the serosal surface. The immunohistochemical analysis showed that the tumor was positive for CD10 and negative for smooth muscle markers. 

CD10 is a surface neutral endopeptidase which was described in immature lymphoid cells [12] and later was found to be a sensitive diagnostic marker for a majority of endometrial stromal sarcomas [13]. This marker is also found to be expressed in high-grade lesions such as leiomyosarcoma [14]; however, as previously mentioned above, the present case tested negative for myogenic markers. 

Kurihara et al. recommended a new terminology and classification of undifferentiated endometrial sarcomas (nonlow-grade endometrial stromal sarcomas) based on nuclear pleomorphism [15]. They divided these sarcomas in two groups: undifferentiated endometrial sarcoma with nuclear uniformity (UES-U) and undifferentiated endometrial sarcoma with nuclear pleomorphism (UES-P). They showed that UES-U shares some molecular and immunohistochemical characteristics with low-grade endometrial sarcoma while considerable differences were observed between UES-P and low-grade tumors.

Based on the infiltrative growth pattern, increased mitotic figures, necrosis, and marked cellular atypia we have classified this tumor as undifferentiated endometrial sarcoma with osteoclast-like giant cells. Using the Kurihara classification this tumor should be classified as type 2 tumor or undifferentiated endometrial sarcoma with nuclear pleomorphism and osteoclast-like giant cells. The histological differential diagnosis in such cases is broad and includes: endometrial carcinosarcoma with osteoclast-like giant cells as described by Amant et al. [16] (positivity of the epithelial components for EMA and Pancytokeratin), undifferentiated metastatic carcinoma (AE1/3 positivity), primary uterine carcinomas, epithelioid leiomyosarcoma (myogenic markers positivity) or metastatic gastrointestinal stromal tumors [17, 18] (CD117 positivity). Mixed Mullerian tumors were shown to resemble endometrial stroma tumors with positivity for CD10, WT-1, and pancytokeratin as well as variable expression of estrogen, progesterone, androgen receptors and myogenic markers [19]. The case we present in this paper was extensively sampled and shown to be completely negative for pancytokeratin (AE1/3), estrogen and progesterone receptors as well as myogenic markers (desmin and myogenin) thus ruling out the diagnosis of mixed Mullerian tumor with stromal sarcomatous overgrowth, the negativity for myogenic markers also rules out epithelioid leyomiosarcoma. The negativity of CD117 (c-kit) also ruled out the possibility of malignant gastrointestinal stromal tumor with osteoclast-like giant cells [18].

Microscopically, the tumor was infiltrating more than two thirds of the myometrium without detectable extension into the endocervical stroma or endocervical glands. The bilateral adnexa were free of tumor and the pelvic wash was negative for malignancy. The tumor was staged as pT1b(Ib), N0 and M0. Although the regional lymph nodes were negative, extensive lymphovascular permeation was present. The tumor disseminated to the peritoneal cavity and lungs causing a vascular thrombosis which led to bowel ischemia and subsequent peritonitis and sepsis.

To the best of our knowledge, this is the first report of a case showing an undifferentiated endometrial sarcoma with nuclear pleomorphism associated with presence of osteoclast-like giant cells and no true osteochondromatous differentiation, which further expands the spectrum of this rare uterine neoplasms. We believe that the osteoclast-like giant cells are most probably from stromal or peripheral blood origin, attracted by the presence of specific cytokines released by the tumor. Although the tumor was found to have a low pathologic stage, it proved to be a neoplasm with highly aggressive behavior and rapid progression. Perhaps this morphologic variant signifies a poor prognosis and requires a more aggressive treatment plan.

## Figures and Tables

**Table 1:** Immunohistochemical stains used in this study and tumor staining results.

Antibody	Clone	Source	Dilution	Company	Staining results
Caldesmon	h-CD	Mouse	1 : 400	Dako	Negative
Calretinin	5A5	Mouse	RTU^1^	Novocastra	Negative
CD10	56C6	Mouse	RTU	Leica	Positive, 3+ in >95% cells
CD56	CD564	Mouse	RTU	Leica	Negative
CD68	514H12	Mouse	RTU	Leica	Positive in osteoclast-like giant cells
AE1-3	AE1/AE3	Mouse	1 : 200	Covance	Negative
DES	D33	Mouse	1 : 1 K	Dako	Negative
HCG	Polyclonal	Rabbit	1 : 2 K	Dako	Rare giant cells were positive
INHIBIN-a	R1	Mouse	1 : 5	Serotec	Positive
Myf-4	LO26	Mouse	1 : 50	Novocastra	Negaive
MYO	Polyclonal	Rabbit	1 : 2 K	Dako	Negative
S-100	Polyclonal	Rabbit	RTU	Leica	Negative
WT-1	WT49	Mouse	RTU	Leica	Positive, 40%–50% tumor cells
ER	1D5	Mouse	1 : 50	Dako	Negative
PR	Pgt636	Mouse	1 : 500	Dako	Negative
CD117	Polyclonal	Rabbit	1 : 400	Dako	Negative

^1^RTU:  Ready to use.
